# Living Lithic and Sublithic Bacterial Communities in Namibian Drylands

**DOI:** 10.3390/microorganisms9020235

**Published:** 2021-01-23

**Authors:** Steffi Genderjahn, Simon Lewin, Fabian Horn, Anja M. Schleicher, Kai Mangelsdorf, Dirk Wagner

**Affiliations:** 1 GFZ German Research Centre for Geosciences, Section Geomicrobiology, Telegrafenberg, 14473 Potsdam, Germany; Simon.Lewin@zalf.de (S.L.); fabian.horn@gfz-potsdam.de (F.H.); dirk.wagner@gfz-potsdam.de (D.W.); 2 GFZ German Research Centre for Geosciences, Section Organic Geochemistry, Telegrafenberg, 14473 Potsdam, Germany; aschleic@gfz-potsdam.de; 3 GFZ German Research Centre for Geosciences, Section Anorganic Chemistry, Telegrafenberg, 14473 Potsdam, Germany; kama@gfz-potsdam.de; 4 Institute of Geosciences, University of Potsdam, 14476 Potsdam, Germany

**Keywords:** lithobiont, intracellular DNA, extracellular DNA, weathering, dryland, rock

## Abstract

Dryland xeric conditions exert a deterministic effect on microbial communities, forcing life into refuge niches. Deposited rocks can form a lithic niche for microorganisms in desert regions. Mineral weathering is a key process in soil formation and the importance of microbial-driven mineral weathering for nutrient extraction is increasingly accepted. Advances in geobiology provide insight into the interactions between microorganisms and minerals that play an important role in weathering processes. In this study, we present the examination of the microbial diversity in dryland rocks from the Tsauchab River banks in Namibia. We paired culture-independent 16S rRNA gene amplicon sequencing with culture-dependent (isolation of bacteria) techniques to assess the community structure and diversity patterns. Bacteria isolated from dryland rocks are typical of xeric environments and are described as being involved in rock weathering processes. For the first time, we extracted extra- and intracellular DNA from rocks to enhance our understanding of potentially rock-weathering microorganisms. We compared the microbial community structure in different rock types (limestone, quartz-rich sandstone and quartz-rich shale) with adjacent soils below the rocks. Our results indicate differences in the living lithic and sublithic microbial communities.

## 1. Introduction

In desert and dryland regions primary production is low due to lack of water, nitrogen and/or phosphorus limitation [1,2,3] but, nevertheless, microorganisms are considered to be the key drivers of ecosystem processes in these environments. Microorganisms of arid regions have to withstand harsh conditions such as rare and irregular precipitation, frequent alternation of cold and hot temperature extremes, high salinity, UV-radiation and fast winds [4]. As a consequence, they have developed adaptation mechanisms with respect to desiccation, osmotic stress, thermodynamics and radiation [1]. One survival strategy is the formation of resting stages such as endospores, exospores and cysts [5]. The transformation from vegetative cells into metabolically dormant state occurs in response to unfavourable external influences such as water scarcity and/or high salt concentration. Additionally, DNA repair mechanisms enable microorganisms to resist strong radiation [1]. Furthermore, microorganisms are able to perform strategies to reduce environmental stress by colonizing refuge niches that provide a protective environment by shielding against stress as solar radiation, re-hydration and desiccation, and temperature variations [6]. Dryland microbial niches for instance are soil crusts or rocks [1]. The porous interior of rocks forms a suitable niche for lithobionts to survive due to better nutrient availability and constant water supply [6]. According to Wierzchos et al. [4] lithobiotic microorganisms can inhabit the rock surface (epilithic), the rock underside in contact with the soil (hypolithic) or they can grow inside the rock (endolithic). Hypolithic communities comprise various groups of heterotrophic bacteria from the phyla Actinobacteria, Acidobacteria, Proteobacteria and Bacteroidetes and phototrophic Chloroflexi [2,7,8]. Bacterial lineages identified in dryland soils are Firmicutes, Chloroflexi and Gemmatimonadetes [1,7] and archaeal representatives are mainly formed by Thaumarchaeota [2] and Halobacteria [9,10,11]. In desert environments the hypolithic microbial community can be dominated by Cyanobacteria [12]. Ramond et al. [13] describes the relevance of hypoliths in the Namib Desert gravel plaints and stress their importance of hot desert productivity via their capacity for N-fixation.

Microorganisms on rock surfaces, in cracks or in pores sometimes form biofilms that influence the breakdown of rocks [14]. Microbial-driven rock weathering has been observed around the world, for example in hot and cold deserts [15,16,17] or in semi-arid to temperate regions [18,19] and the importance of microbially-promoted mineral weathering for nutrient acquisition was reported [20]. Therefore, modern subaerial endolithic structures of extreme environments have the potential to provide insights into early principles of organo-mineral interactions and soil formation from hard rocks [6].

Due to the low microbial biomass and the specific chemical characteristic of rocks DNA extraction is technically challenging [21]. Using commercial environmental DNA extraction protocols, a mixture of living, dormant and dead cells of microorganisms, or rather extracellular DNA (eDNA) and intracellular DNA (iDNA), is extracted [22].

Extracellular DNA from microorganisms is ubiquitous in sediments and soils, in marine and freshwater ecosystems, and in biofilms [7,23,24]. Often, eDNA comprises the largest proportion of total environmental DNA [25] and has been considered to bias microbial community studies [26]. Extracellular DNA may originate from the lysis of dead cells [27], autolysis and active secretion systems of living cells, allochthonous input of biogenic matter, association with extracellular membrane vesicles or horizontal gene transfer [23,28,29]. Free DNA in soils is degraded by microorganisms, but fractions of it can persist for a longer time in the sediment due to adsorption onto mineral or organic matrices [30,31]. Therefore, discrimination of eDNA and iDNA can be a valuable attribute in studying environmental diversity, ecology and functionality.

This manuscript addresses microbial colonization of rocks and soils in an arid landscape. We studied different types of deposited rocks and the underlying soils in central southern Namibian drylands. A culture-based approach was used for understanding the physiological potential of isolated organisms, but do not necessarily provide comprehensive information on the microbial community structure. Therefore, we investigated the composition of lithobiontic and soil microbial communities using the eDNA and iDNA extraction method with special interest in microbial induced rock weathering processes. This comparison allowed the identification of the lithic microbial key community occurring in rocks as a form of terrestrial colonization in drylands. In addition, the analysis and distinction between eDNA and iDNA could indicate potentially viable cells.

## 2. Methods

### 2.1. Study Site and Sampling

**Figure 1:**
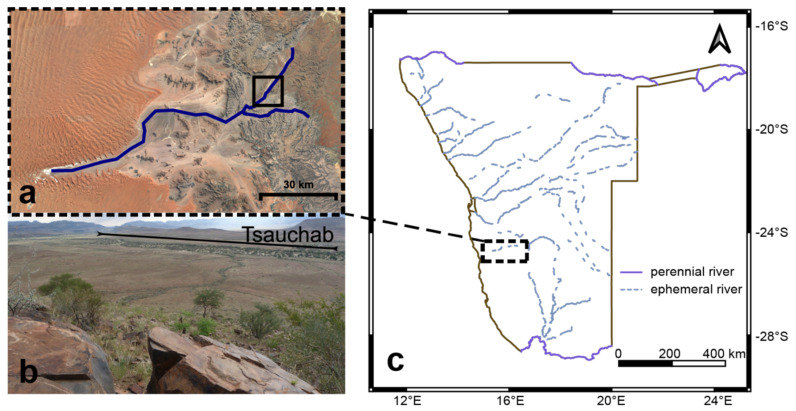
Sampling site at the Tsauchab River (Namibia): (**a**). Satellite image of the Tsauchab (Google Earth V 7.3.2 (2018). sources: Image Landsat/Copernicus). (**b**). View of the Tsauchab River valley (Tsauchab River Camp 24°26′41.5′′ S, 16°10′57.6′′ E). (**c**). Map of Namibia with indication of the study site at the Tsauchab River. Quantum GIS 3.10 was used to produce the map (http://www.qgis.org).

Sampling took place in March 2017 during a field campaign in cooperation between the German Research Centre for Geosciences (GFZ), Senckenberg am Meer—German Centre for Marine Biodiversity Research (DZMB), the University of Oldenburg—Institute for Chemistry and Biology of the Marine Environment (ICBM), and the Technical University of Munich. This work was part of the interdisciplinary project “Signals of climate and landscape change preserved in southern African GeoArchives” within the SPACES program (Science Partnerships for the Assesment of Complex Earth System Processes) initiated by the German Federal Ministry of Education and Research (BMBF). The starting point for sampling was the Tsauchab River Camp located in the Tsauchab River Valley surrounded by the Naukluft and Tsaris Mountains in central Southern Namibia. Spring floods with high sediment loads formed terraces, slopes and fills of young age [32]. A high frequency of drastic floods took place during the Little Ice Age [33]. The ancient Tsauchab river system runs along the Sesriem Canyon and ends in the Namib Desert at Sossusvlei (Figure 1). Nowadays, the Tsauchab catchment (4000 km^2^) receives a maximum of 150–200 mm precipitation annually [3] and has comprises a sparse vegetation such as dwarf shrubs savannah including scarce wood- and grasslands typical for savannah landscapes [34]. The clay mineral composition of the Tsauchab region was described by Heine *et*. *al*. [35]. Illite was the most prominent mineral, followed by chlorite and palygorskite. Moreover, a strongly variable smectite and kaolinite content was measured. The geology of the catchment area consists of limestone, sandstone and shales [3].

### 2.2. Sampling and Sample Characteristics

Three different rock types (limestone, quartz-rich sandstone and quartz-rich shale) were collected nearby the Tsauchab River Camp (24°26′37′′ S, 016°10′31′′ E). For DNA extraction, we sampled three different limestones, three quartz-rich shale and two quartz-rich sandstones. The size of each rock varied between 15–20 cm length and 10–15 cm thickness. Samples were taken from the core, the subsurface and the surface of each rock. The corresponding layer (0–1 cm) of the underlying soils was sampled in biological triplicate. For sampling list see Supplement Table S1.

The total organic carbon (TOC) content of each sediment samples was measured according to DIN ISO 10694 by the Potsdamer Wasser und Umweltlabor GmbH and Co. KG, Potsdam, Germany.

### 2.3. Mineralogy of Rock Samples

To study the mineral composition of the ground powder rock samples X-ray powder diffraction (XRD) analyses were done using a PANalytical Empyrean XRD with a theta-theta goniometer, Cu-Kα radiation (λ = 0.15418 nm), automatic divergent and anti-scatter slits, and a PIXcel3D detector. All measurements were conducted at 40 kV and 40 mA from 4.6 to 85.0° 2θ with a step size of 0.013° and a step time of 60 s. The samples were crushed and powdered to a grain size of <62 μm.

### 2.4. Preparation and Subsampling of Rocks

All rock samples were separated by their visual appearance and their mechanical response into inner core material, subsurface material and surface material. The rock surface was washed with 0.12 M sodium phosphate buffer (pH 8), incubated and shaken for 10 min. With this method, non-permanently attached organisms and particles were detached. The rock sample preparation was performed inside a laminar flow bench, placing the rock sample in a cleaned and autoclaved stainless-steel box. The stainless-steel box and rock processing equipment was specially designed for sterile preparation and sampling of the rocks at the GFZ. The rocks were split into half. One part was used to obtain the surface and subsurface zone. When applying mechanical power, these parts randomly fell off in small fragments or peeled off as gravel or sand. The subsurface is defined as the layer between core and surface fraction. These parts typically burst away as continuous slices along distinct borders, which were targeted with a chisel. In order to obtain the inner core fraction, rocks were UV irradiated (254 nm) for 30 min from two orientations within the clean bench. Thus, microorganisms attached to the surface were degraded and should not penetrate into the freshly exposed core zone. The UV irradiated surface material was removed from the core sample by chiselling. Finally, all rock fractions were crushed between two ridged stainless-steel discs in a hollow steel cylinder. Crushed rock material was then pulverized using a mortar and pestle.

### 2.5. Extra- and Intracellular DNA Extraction

The extraction of eDNA and iDNA from sediment and pulverized rock samples was performed after [22]. Both iDNA and eDNA were separated from the sample matrix, while organic components and downstream inhibitors were removed by binding to the highly cross-linked polymer polyvinylpolypyrrolidone (PVPP). Therefore, 8 g of sample material was mixed with 0.6 g PVPP and 7 mL sodium phosphate (NaPNa_2_PO_4_) buffer in a sterile 50 mL falcon tube, allowed to rest on ice for 1 min and then shaken in a centrifuge for 5 min at 150 rpm. The samples were cooled on ice for 3 min and the shaking procedure was repeated. The resulting slurry was centrifuged for 10 min at 500× *g*. This procedure was repeated three times, with the pellet re-suspended in 3.5, 2 and 1.5 mL NaPNa_2_PO_4_. The pellet contained the iDNA released from the intact cells while the eDNA remained in the clear supernatant. The supernatant was pooled and divided into three technical replicates and centrifuged for 1 h at 4643× *g*. Finally, the supernatant was purified from residual cells using a 0.2 μm syringe filter and kept on ice for further processing. The pellet was washed with 1 mL NaP to obtain the iDNA. The cell lysis was performed using a Power Soil^®^ DNA Isolation Kit (Mo Bio Laboratories Inc., Carlsbad, CA, USA) including heating up to 70 °C, bead beating in presence of SDS and purification from cellular components and inhibitors. Within the last step i- and eDNA were purified and concentrated. The iDNA and eDNA solutions were shaken together with guanidine hydrochloride (GuaHCl, 6M, 4× volume of extract) and silica particles (ρ: 1.3 g mL^−1^) for 45 min at 175 rpm followed by incubation on ice for 10 min and centrifugation for 10 min at 4643× *g*. The supernatant was discarded and the silica pellets were washed with 70% ethanol buffer. After drying the pellets, the DNA was eluted in TE buffer at 50 °C for 10 min at 250 rpm. No sample template was used as a negative control for either the eDNA or iDNA extraction. A PCR was carried out to check the contamination. The negative controls revealed no visible PCR product on the agarose gel.

### 2.6. Preparation for Next-Generation Sequencing

The microbial community of three rock types and of rock covered soil was examined by 16S-rRNA MiSeq amplicon sequencing. Each 16S rRNA amplicon originating from an individual DNA sample had been previously assigned to a unique barcode combination using tagged primer pairs. The primer sequence consisted of a 6-bp tag and spanned the V4 region of Bacteria and Archaea. The universal primer pair 16S-515F (5’-GTGCCAGCMGCCGCGGTAA-3′) and 16S-806R (5’-GGACTACHVHHHTWTCTAAT-3′) [36] was chosen for the barcode PCR. The 25 μL PCR reaction mix consisted of polymerase buffer 1×, MgCl_2_ (3 mM), and ultra-pure dNTPs (0.2 mM), optitaq polymerase 0.05 U μL^−1^ manufactured by Bioline GmbH, Luckenwalde, Germany) and 1μL of each primer (5 µM), and 2.5μL of template. The mix was filled up to 25μL with PCR-clean water (MO BIO Laboratories, Inc., Carlsbad, CA, USA). The touchdown PCR protocol started with an initial denaturing phase of 5 min at 95 °C, followed by 10 cycles of 30 s at 95 °C, 30 s starting at 51 °C (decrease of 1 °C each cycle) and 30 s at 72 °C. After completion of the first phase 20–30 cycles were subsequently performed using the same time intervals and temperatures. Finally, temperature was hold for 7 min at 72 °C followed. This touch down PCR protocol avoids excessive amplification cycle of unspecific products, increases specificity, selectivity and yield [37]. In order to obtain a sufficient amount of PCR product, three (soil) or four (rock) reactions from the same template where pooled before purification. PCR products were purified using the magnetic bead-based Agencourt AMPure^®^ XP-Kit and, finally, eluted with 45 μL PCR-grade H_2_O (MO BIO Laboratories, Inc., Carlsbad, CA, USA) preheated to 55 °C. The final DNA concentration was quantified by a Qubit Fluorometer 2.0 (InvitrogenTM, Thermo Fisher Scientific, Waltham, MA, USA). The samples were sent to the Illumina MiSeq platform (Eurofins Genomics, Ebersberg, Germany). Data were obtained as raw FASTQ files and deposited into the European Nucleotide Archive (sample accession: ERS3207779—ERS3207809).

### 2.7. Processing Next-Generation Sequencing Data

The Illumina paired-end sequencing technology generates reads from DNA fragments that were merged using the software PEAR [38]. Nucleotide sequences were orientated, trimmed and low-quality sequences were filtered using the preprocessing tool Trimmomatic [39]. All chimeras were removed and sequences were clustered into operational taxonomic units (OTUs) using the QIIME pipeline. The taxonomic classification was assigned by the SILVA database (Version 128) [40] with a similarity threshold of 97% using the QIIME open-source software package and by choosing the open-reference OTUs [41]. The sequencing data set was filtered on mitochondrial and chloroplast sequences and a cut-off, with less than 1% over the entire abundance, has been set. The dataset was checked by NMDS and rarefaction curve analysis. To rule out contaminants positive and negative controls were sequenced and analysed together with all other samples. The positive control was 100% assigned to *Escherichia-Shigella*. The negative PCR control showed no significant contamination and was filtered away due to the low quality of the reads.

### 2.8. Statistics

Absolute read counts were transformed to relative abundances to standardize the data and to account for different sequencing depths. Diversity indices were calculated based on the total counts of all OTUs using the Past 4.03 software [42]. The taxonomic abundance across samples was visualized in R by the ggplot2 v.3.1.0 package [43]. The heatmap was created in R using the ‘pheatmap’ package [44]. For the creation of the heat map, relative abundances are represented on class level and with more than 1% abundance within all samples.

### 2.9. Isolation of Bacteria

Isolation of bacteria from rock samples was performed on two different media. R2A is a widely used low in nutrients medium for growth of general facultative heterotrophic aerobic organisms, containing (L^1^) 0.5 g C_6_H_12_O_6_, 0.5 g yeast extract, 0.5 g caseinhydrolysat, 0.5 g starch, 0.39 g C_3_H_3_NaO_3_, 0.3 g K_2_HPO_4_, 0.0249 g MgSO_4_ and 15 g agar. In addition, a sucrose salt medium (SSM) was used to screen for strains adapted to the lithic niche or mineral solubilizing organism [45,46]. The media SSM contains 10.0 g C_12_H_22_O_11_, 0.5 g yeast extract, 1.0 g NH_4_SO_4_, 2.0 g K_2_HPO_4_, 0.1 g NaCl, 0.5 g CaCO_3_, 0.5 g MgSO_4_ and 18.75 g agar. The final pH of both media was adjusted to 7.2 using NaOH or HCl.

A total of 10 g of crushed and pulverized rock samples were suspended in 250 mL physiological NaCl solution in an Erlenmeyer flask and shaken at 200 rpm. After 30 min, 24 h and 7 d, 1 mL of the suspension was plated on R2A and SSM and incubated at 28 °C. If single colonies were visible, these were repeatedly transferred to fresh plates until cultures of uniform morphology were obtained. Purity of the strains was confirmed by light microscope examination.

### 2.10. Identification of Microbial Isolates

Genomic DNA was extracted from a colony of each pure culture. A minimal amount of the culture was dipped and suspended with a sterile pipette tip into 50 μL TE buffer. The suspension was incubated at 90 °C for 10 min and immediately transferred on ice for 2 min. After a final centrifugation step at 13,000× *g* for 10 min 1µL of the supernatant was added to a touch down PCR using the bacterial universal primer pair 16S-27F (5′-AGAGTTTGATCCTGGCTCAG-3′) and -1492R (5′-GGTTACCTTGTTACGACTT-3′) [47]. The cycling program involves two separate phases starting with the touchdown phase. After an initial denaturing phase of 5 min at 95 °C, 10 cycles of 30 s at 95 °C, 45 s starting at 64 °C (decrease of 1 °C each cycle, down to 54 °C) and 60 s at 72 °C followed. After completion of the first phase 30 cycles were subsequently performed: 60 s at 95 °C, 30 s at 54 °C and 60 s at 72 °C and, finally, temperature was held for 7 min at 72 °C. The PCR mix (25 µL) included: Polymerase buffer 1× (Quiagen, Hilden, Germany), Optitaq Polymerase 0.05U μL^−1^ (Roboklon GmbH, Berlin, Germany), MgCl_2_ 3 mM and dNTPs 0.2 mM manufactured by Bioline GmbH, Luckenwalde (Germany), forward and reversed primer (0.5 µM), template as required and PCR-grade H_2_O (19.5—V_template_). The PCR products were checked by gel electrophoresis and purified using the Hi Yield^®^ PCR (Süd-Laborbedarf GmbH, Gauting, Germany) fragments extraction kit. 0.2–1 μg of the purified PCR fragment were sent to Eurofins GATC Biotech GmbH for Sanger sequencing on an ABI 3730xl DNA analyser system. The 16S rRNA sequences were compared by BLAST [44,48] to known sequences in GenBank (www.ncbi.nlm.nih.gov).

### 2.11. Quantification of 16S Gene Copy Numbers

The quantification of the bacterial 16S rRNA gene was based on the primers 16S-341F (5′-CCTACGGGAGGCAGCAG-3′) and 16S-534R (5′-ATTACCGCGGCTGCTGG-3′). Gene copy number were analysed in triplicate using a thermal cycler (CFX ConnectTM Real-Time PCR Detection System, Bio-Rad Laboratories, Irvine, CA, USA) in combination with the BioRad CFX Manager Software. Each 20 µL PCR essay contained KAPPA Hifi SYBR Mix 1× (Qiagen, Hilden, Germany) forward and reverse primer 0.2 μM, PCR-grade H_2_O 5.92 μL, and 4 µL template. To generate a standard curve, known dilutions (10^1^–10^7^ gene copies) of the target fragments amplified from *Bacillus subtilis* (for bacteria) were used. Melting curve analysis was conducted at the end of each run to identify nonspecific amplification of DNA. The efficiency (>90%) was calculated based on the standard curve using the BioRad CFX.

## 3. Results

### 3.1. Total Organic Carbon of Soil and Mineral Composition of Rocks

Total organic carbon (TOC) values of the soils were measured in triplicates. The TOC values range from 0.09 to 0.21%. The average TOC value in limestone was 0.18%, in quartz-rich sandstone 0.15% and in quartz-rich shale 0.13%.

The obtained XRD results revealed that the surface material, the subsurface material and the inner core material of the limestone is composed of dolomite and minor plagioclase, whereas the three quartz-rich sandstone separates contain mostly quartz and minor plagioclase. The quartz-rich shale separates show a variety of minerals, including quartz, mica (illite/muscovite/biotite), chlorite/kaolinite, calcite and plagioclase. Distinct mineralogical differences in the surface, subsurface and core material of the individual rock types have not been observed, indicating no strong weathering within the rocks. The XRD patterns of the different rocks are displayed in Supplement Figure S1.

### 3.2. Abundances of Microorganisms

The bacterial gene copy numbers were measured in soil and all three subsections of limestone, quartz-rich sandstone and quartz-rich shale stone (inner core, subsurface, surface). All rocks showed very low abundances in eDNA and iDNA ranging from 10^1^ to 10^2^ gene copies g^−1^ rock powder (Supplement Figure S2). In soils a higher abundance between 10^5^ and 10^6^ gene copies g^−1^ was observed. In all soil samples the abundance of eDNA was higher than iDNA (Supplement Figure S2).

### 3.3. Microbial Composition of the eDNA and iDNA Pools in Rocks

**Figure 2:**
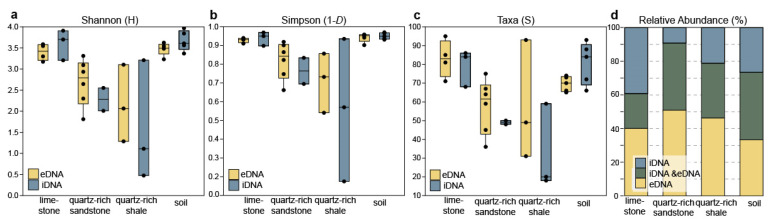
Evenness, diversity and OTU distribution. Differences between eDNA (yellow) and iDNA (blue). Shannon evenness (**a**) and diversity (Inverse Simpson) indexes (**b**) observed in quartz-rich sandstone, quartz-rich shale, limestone and soil. Number of taxa (S) **(c)** and relative abundance (%) of the OTUs in the e- and iDNA pool as well as the shared OTUs (dark green) from both DNA pools (**d**).

The microbial communities of the three different rock types were analysed. In the beginning, the rocks were separated into three intersections (core, subsurface, surface) and three soil sample (1 cm below the rock) per site. Due to the small recovery of DNA, the results of all intersections of each rock were combined to run statistical analyses of the microbial community. DNA concentration measurements using a Quibit fluorometer were not possible, but gene copy numbers are shown in Supplement Figure S2. In total, 340 OTUs were assigned to a taxonomy and remained after quality filtering in the dataset. The microbial diversity of limestone in both DNA pool is higher than in quartz-rich sandstone and shale rocks. In addition, soil samples show a high diversity in iDNA and eDNA. The variation within the quartz-rich samples is higher than in limestone or soil samples. The Shannon and Simpson indices are shown in Figure 2a,b. The number of taxa (including eDNA and iDNA) decreased from limestone over quartz-rich sandstone to quartz-rich shale. The variation within quartz-rich shale samples is high (Figure 2c). The separation of eDNA from iDNA showed clear differences between the two pools with varying proportions of unique and shared (overlap) OTUs in the different rock samples (Figure 2d). Limestone showed the same ratio of unique OTUs in the iDNA or eDNA (1:1) pool with minor proportion of OTUs shared with both pools. Contrastingly, a reduced number of unique OTUs within iDNA compared to eDNA was present at quartz-rich sandstone (6:1) and quartz-rich shale (2:1) (Figure 2d). Shared pools of e- and iDNA were different in rocks: quartz-rich shale 40%, quartz-rich sandstone 33% and limestone 21% (Figure 2d).

**Figure 3:**
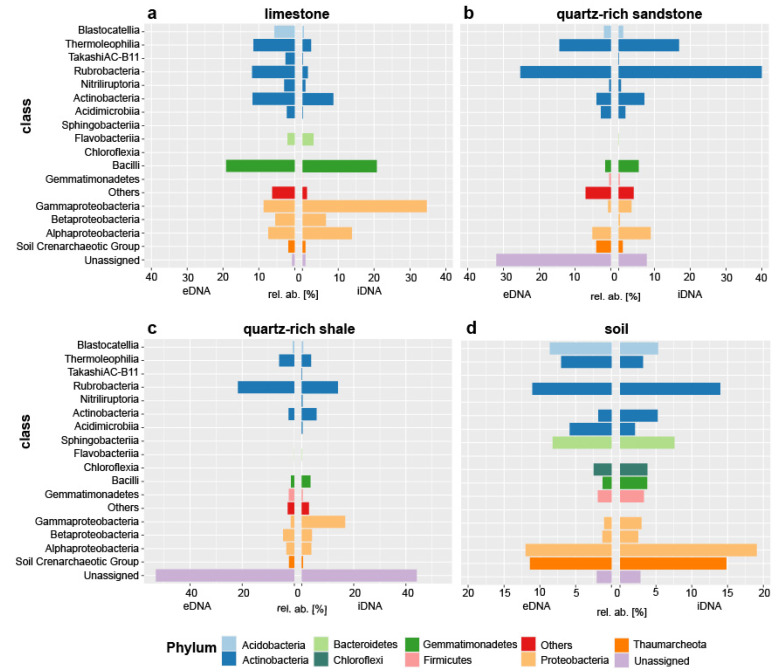
Comparison of the relative abundance. Abundance of eDNA and iDNA from different rock types (limestone, quartz-rich sandstone and quartz-rich shale) and the adjacent soil at class level. Microbial classes with an abundance less than 1% are summarized in others. Color codes assigned to phylum and are indicated below.

Differences between eDNA and iDNA on class level are shown in Figure 3. Absolute read counts were transformed to relative abundances in order to standardize the data. Detailed taxonomic information is assigned to the relative abundance of OTUs within the rock and soil microbial community (Figure 3). Limestone rocks were dominated by Proteobacteria, Actinobacteria and Firmicutes. Proteobacteria was dominating the eDNA pool with a relative abundance of 57%, followed by Firmicutes with 22% and Actinobacteria with 14% (Figure 3a). In contrast, the relative abundance of the dominated phyla in the iDNA pool was different Actinobacteria (43%), Proteobacteria (23%) and Firmicutes (20%) (Figure 3a). A more detailed distinction between eDNA and iDNA was visible when looking at the actinobacterial and proteobacterial composition in limestone separately. *Rubrobacter* (46% of all Actinobacteria) and Gaiellales (17%) were the most abundant Actinobacteria in the iDNA pool of limestone. In contrast, the eDNA pool was dominated by Propionibacterium (31%), *Rubrobacter* (22%) and *Solirubrobacter* (14%). The composition of Proteobacteria was different in the eDNA and iDNA pool. *Pseudomonas* (33% of all Proteobacteria), *Enhydrobacter* (24%) and *Methylobacterium* (11%) made up the majority of the proteobacterial eDNA. Whereas sequences of the iDNA were mainly assigned to the genera *Enhydrobacter* (25% of all Proteobacteria), *Pseudomonas* (15%), *Massilia* (15%), *Sphingomonas* (10%) and *Acinetobacter* (10%) (Figure 3a).

Quartz-rich sandstone rocks were dominated by *Actinobacteria* (Figure 3b). The majority of actinobacterial sequences belonged to the classes Rubrobacteria (eDNA 42%, iDNA 27%) and Thermoleophilia (eDNA 18%, iDNA 15%). Furthermore, Alphaproteobacteria (9%), Actinobacteria (7%), Bacilli (6%) were present in the eDNA (Figure 3b). The relative abundance of these classes was higher than in the iDNA. Alphaproteobacteria was dominated by the genera *Craurococcus* and *Bacilli* were dominated by the genus *Stapyhlococcus*. Unassigned sequences made up 34% of the iDNA in quartz-rich sandstone (Figure 3b).

Quartz-rich shale rocks were dominated by unassigned sequences (eDNA 45% and iDNA 54%) (Figure 3c). Additionally, a high proportion of the classes Gammaproteobacteria (17%) and Rubrobacteria (14%) were found. In contrast, the iDNA pool was composed of Gammaproteobacteria (1%) and Rubrobacteria (27%). Furthermore, actinobacterial sequences related to the classes Thermoleophilia and Actinobacteria ranged from 2 to 6%. The genus Acinetobacter dominated the abundance of Gammaproteobacteria in the eDNA of quartz-rich shale (Figure 3c).

### 3.4. Main Differences in the Microbial Composition between Rock and Soil

**Figure 4:**
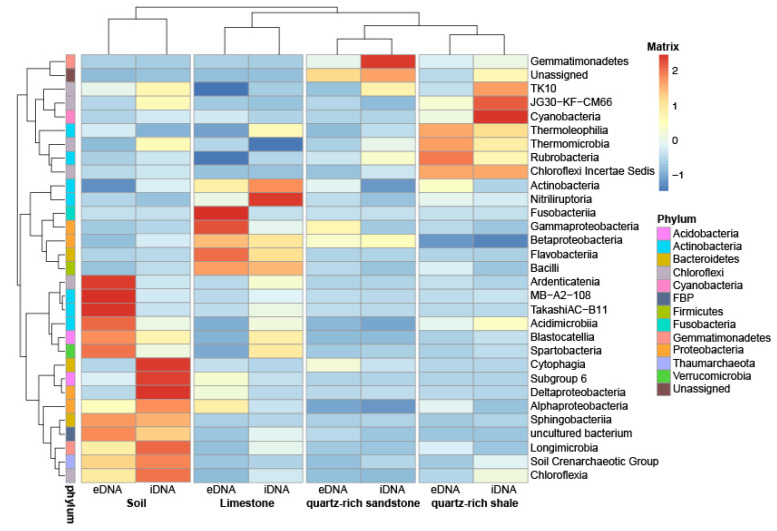
Heatmap clustered based on the microbial community similarity. Heatmap of microbial community composition with cluster analysis on class level. The colour intensity in each panel shows the percentage in a sample, referring to colour key at the right. Identification of shared OTUs between different rocks (quartz-rich shale, quartz-rich sandstone and limestone) and soil samples on taxonomic distribution. Different phyla on the bottom are indicated via color-coding.

In soil 40% of eDNA- and iDNA OTUs were shared (Figure 2d). In contrast, when comparing the DNA of rocks and the soil below, only a small amount of OTUs was shared. Specifically, this means that between 8 and 16% were shared in the eDNA and between 6 and 7% in iDNA. The sublithic soil communities from the three different rock type’s limestone, quartz-rich sandstone and quartz-rich shale were similar and the results were therefore compiled in Figure 3d. Thaumarcheaota (exclusively Soil Crenarcheoatic Group) and Acidobacteria (mainly Blastocatellia) were found in soils in both eDNA and iDNA pools (Thaumarcheaota 15% vs. 16% and Acidobacteria 11% vs. 6%, Figure 3d). Both were nearly absent in rocks. The actinobacterial community differed in composition between soil and rocks. The resulting 31 most abundant classes of the rocks and soils are determined and summarized in a heat map (Figure 4). These OTUs were distributed over 12 phyla. In soil Rubrobacteria occurred in high relative abundance in both eDNA (15%) and iDNA (15%) pools and were the most abundant Actinobacteria in quartz-rich rock and in soil. In soil a higher proportion of Actinobacteria assigned to Acidimicrobiia, MB-A2-108 and Takashi AC-B11 were detected than in rock samples (Figure 4). In contrast to soil, the relative abundances of Actinobacteria assigned to Corynebacteriales, Geodermatophilaceae, Micrococcales, Propionibacteriales and Pseudonocardiales were higher in rocks. The relative abundance of Bacteroidetes in soil was higher than in rocks. In soil the phyla Bacteroidetes occurred in the eDNA (11%) and iDNA pool (8%) and were dominated by Sphingobacteriia (especially Chitinophagaceae) (Figure 3 3d and Figure 4). The proportion of Chloroflexi (including mostly Ardenticatenia and Chloroflexia) in soil was higher than in rock samples (Figure 3d). In soil Firmicutes were less abundant. In contrast, a high abundance of Firmicutes was detected in limestone (Figure 4). The proteobacterial community was distinct between soil and rocks (Figure 4). The main differences were the abundance of Alpha- and Gammaproteobacteria. The soil was dominated by Alphaproteobacteria in both e- and iDNA pools (mainly Rhizobiales and Sphingomonadales) whereas limestone and quartz-rich shale were dominated by Gammaproteobacteria (mainly Pseudomonadales), especially the eDNA pools ( Figure 3; Figure 4). A low proportion of Gemmatimonadetes was detected in soil and rocks (Figure 3), while in soil Gemmatimonadetes occurred almost in the same proportion in the e- and iDNA pools (3%/4%). Moreover, Cyanobacteria were detected in very low abundance in soil and rocks (<0.3%) as well as Verrucomicrobia (<1%). Fusobacteria were only detected in low relative abundance in rocks. Additionally, phyla with less than 0.1% of relative abundance were found, such as Euryarchaeota, Armatimonadetes, Chlorobi, Deinococcus-Thermus, FBP, Nitrospirae, Parcubacteria, Planctomycetes, SBR1093 and Saccharibacteria. A high proportion of the rock microbial community is unknown and database search fails to assign a sequence to a specific taxon, especially in quartz-rich shale rock.

### 3.5. Microbial Isolates Recovered from Rocks

Isolated microorganisms with homologous colony morphology were identified by their 16S-rRNA gene using Sanger sequencing. In total, 28 isolates were obtained on R2A and SSM agar plates summarized in Supplement Table S2. Based on combined pairwise alignment isolates of three different phyla (Proteobacteria, Bacillus and Actinobacteria) were identified (Table 1). All three isolates of Proteobacteria originated from limestone and were assigned to Massilia sp. (Burkholderiales) or Microvirga sp. (Rhizobiales). Ten isolates were assigned to Bacillus strains, including *B. axarquiensis*, *B. paralicheniformis* and *B. niacini* in limestones and *B. subtilis* and *B. tequilensis* in quartz-rich sandstones (for details see Supplement Table S2). Isolates of the phylum Actinobacteria included the genera *Streptomyces*, *Protaetiibacter*, *Arthrobacter* and *Kocuria* in limestones, whereas in quartz-rich samples the genera *Microbacterium*, *Arthrobacter*, *Lechevalieria* and *Streptomyces* were isolated (Table 1).

The following percentage of our isolates can also be found in 16S rRNA sequencing data. The organisms are listed below and the percentages of eDNA and iDNA are given in parentheses. For limestone: *Kocuria* (0.99% eDNA/0.94% iDNA), *Streptomyces* (0.09%/0.58%), *Bacillus* (2.6%/2.02%), *Massilia* (0.0%/1.02%) and *Microvirga* (0.25/0.5%). The genus Arthrobacter was not identified but the family Micrococcaceae comprised 0.88% of eDNA and 0.14% of iDNA. In quartz-rich sandstone *Microbacterium* made up 0.20% of eDNA, *Streptomyces* (0.54% eDNA/0.26% iDNA), and *Bacillus* (0.93%/0.75%). The genus *Lechevalieria* was not identified via 16S rRNA sequencing but the family Pseudonocardiaceae comprised 0.85% of eDNA and 1.17% of iDNA.

## 4. Discussion

### 4.1. Characterization of the Lithic Microbial Community in Rocks Using Extracellular DNA and Intracellular DNA Analyses

Environmental DNA consists of DNA from intact and living cells (intracellular DNA) and extracellular DNA representing DNA preserved in soils, sediments and aquatic systems [65]. In general, there is a lack of understanding the origin of eDNA and its dynamics in environmental settings. The possibility of different eDNA sources influences the interpretation regarding microbial abundance and diversity of the present communities [22,66]. However, it is agreed that there are two major sources for eDNA: the active release by intact cells and the passive release from lysing cells [23,24,28]. In addition, active DNA excretion varies from species to species [23] and the preservation of eDNA changes according to different conditions such as clay content, salinity and temperature of the ecosystem [67]. For this reason, the fundamental contribution of eDNA to species richness and interpretation based on total DNA extraction have to be questioned at least in extreme environments. The distinction in eDNA and iDNA can deepen our understanding of living microbial communities and their diversity. Instead of extracting total DNA it comprised greater genetic information about the composition and distribution of microbial communities [26]. To the best of our knowledge, this is the first study to extract e- and iDNA from rocks to characterize the endolithic and hypolithic microbial communities.

The result of high-throughput sequencing of both DNA pools showed that on the one hand unique OTUs in each pool exist and on the other hand, depending on the specific environmental conditions, an overlap of OTUs occurring in both DNA pools (shared pool) was visible (Figure 2d). By looking at the shared eDNA and iDNA pool, we gain a better description of the viable and potentially active microbial communities [7,29] because the identification of eDNA and iDNA of similar origins can represent active cell turnover of the stationary phase. In limestone, 21% of OTUs were shared between eDNA and iDNA (Figure 2d) which were mainly assigned to the phyla Proteobacteria, Actinobacteria and Firmicutes (Figure 3a). The presence in both pools (eDNA and iDNA) indicates that an adapted part of these organisms could probably maintain metabolic activity under the given environmental conditions.

In contrast, higher abundances of unique OTUs in the eDNA pool compared to the iDNA pool could be explained with temporarily favourable living conditions (e.g., water or nutrient availability) or wind erosion processes [16] in the past. The presence of some species only in the eDNA indicated that the environmental conditions have changed accordingly and that some microorganisms are no longer able to survive. Whereas, the majority of cells died and the DNA was released to the environment, some cells form resting stages for future favorable conditions [5]. The eDNA pool of limestone was characterized by high relative abundance of Proteobacteria whereas the iDNA pool was dominated by Actinobacteria. On closer inspection, Pseudomonas, along with the heterotrophic bacteria Acinetobacter and Enhydrobacter, were the main representatives of Gammaproteobacteria in limestone. Pseudomonas is one of the first bacteria to be described in the context of biogenic weathering. Lepleux et al. [68] demonstrated facilitation of highly effective iron release and Puente et al. [14] described solubilization of limestone by Pseudomonas [14]. In addition, the betaproteobacteria genus Massilia was recovered as a bacterial culture (Table 1) as well as detected in the iDNA pool of limestone. Massilia species has been reported to participate in carbonate rock dissolution by acid secretion.

On the contrary, a higher number of unique OTUs in the iDNA pool compared to the eDNA pool can reflect the better adaptation of certain species to the prevailing ecological conditions. For instance, Schulze-Makuch et al. [7] found episodic evidence of biological activity after rainfall events in the Atacama Desert. However, no conclusions about the activity status of the respective microorganisms can be drawn based on 16S rRNA. The major representatives of Actinobacter in limestone were *Rubrobacter* and Gaiellales. Both occurred especially in the iDNA pool indicating their intact and viable stage. *Rubrobacter* is common to desert edaphic and lithic environments [69,70] and is identified as a predominant colonizer of granite in an extreme continental climate [71]. *Rubrobacter* are heterotrophic and known to be resistant to hot temperatures, dehydration and radiation [72]. This resistance is based on the ability to produce compatible solutes [73]. They further can be involved in biofilm formation and mineral precipitation on rock surfaces of lime- and sandstone [69]. Additionally, several actinobacerial isolates could be identified from limestone, e.g., Arthrobacter or Streptomyces (Table 1). Both genera are important for mineral cycling in soils [49]. In addition, the isolated genus *Kocuria* was already covered from limestone [74]. *Kocuria* was reported to solubilize Si and Al and perform mineral weathering [50].

Moreover, Firmicutes—dominated by *Staphylococcus*—occurred in high frequency in both DNA pools of limestone (Figure 3a). *Staphylococcus* was shown to be involved in mineral weathering processes [50,54,75]. *Bacillus* species have been described previously in the environmental context of saline and arid soils [11,76,77] and rhizosphere association [78]. In this study, ten strains of the genus *Bacillus* were isolated from rock samples (Supplement Table S2). In contrast, sequencing data showed the dominance of non-spore forming Staphylococcus instead of spore-forming *Bacillus*. *Bacillus* spores survive in extreme drought, high UV radiation and ionizing radiation [79]. *Bacillus* is known to produce volatile organic acids like butyric and acetic acid. Secretion of these secondary metabolites leads to the dissolution of surrounding rock substrates [14]. It can be assumed that the slight contribution of endospores to the iDNA pool was not realistically reflected, because the applied iDNA extraction method does not extract DNA from spores [7]. *Bacillus subtilus* are shown to participate in diverse weathering processes [59]. For instance, plagioclase is a mineral component of the quartzite-rich shale rock and was most effectively weathered by *B. subtilis* in culture experiments conducted by Song et al. [59]. Furthermore, some *Bacillus* isolates we have isolated are known to produce biosurfactants [80,81]. Biosurfactants emulsify organic compounds, increase water solubility and make the compounds more accessible to the microorganisms [82].

In addition, some phyla, such as Acidobacteria and Verrucomicrobia, occurred mainly in the limestone iDNA pool indicating their viable stage (Figure 3a). Acidobacteria are commonly found in limestone environments whose role within the ecosystem is still unclear [83,84]. Acidobacteria is found in a variety of habitats and its successful adaptation to harsh soil conditions was demonstrated by Kielak et al. [85]. Apart from the low abundance of Cyanobacteria this agrees with results from arid environments and limestone samples [86,87,88].

Differences in the composition between the eDNA and iDNA pool of quartz-rich shale were observed in the relative abundance of Actinobacteria and Proteobacteria (Figure 3c). Sequences related to Proteobacteria, dominated by Acinetobacter, were highly abundant in the eDNA pool suggesting that living conditions of related species were more appropriate at another time. Acinetobacter were isolated from rocks and soils and were reported to solubilize Al, Si and Fe from rocks and phosphate in the rhizosphere [50,68,89]. Furthermore, Acinetobacter shows a high capacity to induce calcium carbonate precipitation on calcareous rocks [90]. The high proportion of unknown taxonomic groups in quartz-rich shale samples indicates the limitations of the existing sequence database and their undiscovered diversity in such an environment.

Both DNA pools of quartz-rich sandstone were dominated by Actinobacteria mainly represented by *Rubrobacter* and Thermoleophilia (mainly Gaiellales) (Figure 3b,c). The high abundance of *Rubrobacter* in the iDNA pool indicated their presence in a viable stage and could play an active role in mineral precipitation. Thermoleophilia play an important role in carbon decomposition [91]. In addition, we could isolate Actinobacteria from quartz-rich sandstone, e.g., *Microbacterium* and *Kocuria* (Table 1). Both are reported to perform mineral weathering in soils and altered rocks [50,53,54].

*Craurococcus* (Alphaproteobacteria) occurred in equal parts in the eDNA and iDNA pool of quartz-rich sandstone potentially expressing active cell turnover. *Craurococcus* grows heterotrophically under aerobic conditions and is known to reduce nitrate to nitrite [92]. Additionally, the eDNA pool of quartz-rich sandstone was enriched in actinobacterial taxa such as Geodermatophilaceae. Geodermatophilaceae was predominantly recovered from hot and cold arid environments and are often found in calcareous and carbonatic rocks [93]. Geodermatophilaceae have the ability to alternating episodes of calcareous solubilization and precipitation [94] and their resistance to environmental stress (e.g., UV irradiation, dehydration, salinity) is well known [95].

Cyanobacteria occurred in low abundance in limestone or quartz-rich. Water availability plays an important role for the habitability of the endolithic environment but overall, the effect of small rocks on water retention is difficult to assess. Future research will be necessary to identify environmental parameter like moisture or disturbance that might cause the absence of Cyanobacteria in the investigated Namibian drylands.

Comparing eDNA and iDNA of both quartz-rich sandstone and quartz-rich shale rocks about 50% of the OTUs were unique to the eDNA pool (Figure 1d). These could refer to a shift in the composition of the microbial community due to changing environmental factors in the past. A temporary availability of organic material might explain the high abundance of microorganisms in the eDNA pool that may contribute to mineral precipitation (in particular *Staphylococcus*, *Acinetobacter* and *Pseudomonas*). Castanier et al. [96] described an increase of heterotrophic bacteria when the environment is enriched with organic matter until the initial enrichment is consumed. Thus, the heterotrophic bacterial community responds with carbonate precipitation [96]. Furthermore, the analyses of the shared eDNA and iDNA pool suggested that the presence of *Rubrobacter* in particular has a high potential for an active community in quartz-rich rocks. Remarkably, the shared e- and iDNA pool of limestone are the smallest compared to the quartz-rich rocks (Figure 2c,d). In addition, the iDNA pool of limestone harboured 39% of unique OTUs. These could refer to a singular current viable and active community which leads to cyclic formation of eDNA due to natural cell turnover in limestone.

Quartz-rich rocks and limestone are colonized by microbial communities that have the potential for mineral precipitation. Heterotrophic pathways of microorganisms can induce extracellular precipitation of calcium carbonate. In rock samples the number of bacterial taxa capable of producing calcium carbonate is high. In all examined rock types, the presence of *Rubrobacter* indicated their participation in a potentially active microbial community. Moreover, *Staphylococcus* occurred in a viable stage in limestone whereas Acinetobacter and Pseudomonas were present only in the eDNA pool and, therefore, not part of an active community. All the microorganisms just mentioned have been previously reported to be important for biological mineral precipitation [69,97,98].

Culture-dependent studies point to the specialization of taxa in rock colonization in connection with dissolution of rock surfaces, metal solubilization, mineral weathering and production of weathering agents. The isolates represent only a small part of the microbial community due to numerous cultivation limitations. Other organisms could be isolated using different media and growth conditions. To understand and complete the stage of colonization of rocks by lithobionts, future long-term weathering experiments are required. Modern endolithic communities can withstand environmental extremes that make them suitable candidates to assess biogenic weathering processes and development of lithic colonization.

### 4.2. The Sublithic Microbial Community

Complex vegetation cover is reduced with increasing xeric environmental conditions. This leads to a pronounced role of microorganisms in biogeochemical cycling by driving primary productivity and biomass production in desert environments [2,12,99]. The lithic and sublithic microbial communities living in the investigated dryland showed a high diversity, especially in limestone and in soil (Figure 1a,b).

By linking the relative abundance of iDNA and eDNA of rocks and soil, a big difference in the lithic and sublithic community structure was found. The number of shared OTUs (including e- and iDNA) between rocks and soil was low (<25%) (Figure 4). In contrast, previous studies on total DNA of lithic and sublithic communities reporting overlaps of 88% whereby most abundant taxa in the soil were also present in hypoliths but in lower abundance [100]. This large discrepancy can be explained by different extraction methods. In the meantime, the extraction of the entire environmental DNA distorts the view by not separating in dead and viable cell. Here, we could demonstrate that the iDNA and eDNA extraction clarifies the differences between lithic and the sublithic microbial community (Figure 4). A shift in diversity and abundance point to niche separation at the rock-soil margin in drylands.

Changes in the microbial communities between altered rocks and soils were not only determined by the major bacterial classes, but also by a variation at low taxonomic level (Figure 4). The main compositional difference between the soil and lithic microbial communities was a higher relative abundance of Thaumarchaeota (Soil Crenarchaeotic Group), Actinobacteria (mainly Acidimicrobiia and TakashiAC-B11), and Bacteroidetes (mainly Chitinophagaceae) in dryland soils (Figure 4).

Alphaproteobacteria were present in all investigated rocks and soil but Sphingomonadales and Rhizobiales formed the largest proportion in the e- and iDNA pool in soil. In addition, the genus *Microvirga* (Rhizobiales) could be isolated from limestone (Table 1). Microvirga is known to be part of the rhizobia and is capable of symbiotic nitrogen fixation with legumes [64]. Van Goethem et al. [88] described Alphaproteobacteria as a key taxa at the interface between rock and soil and assigned them an important functional role in the microbial community structure. Rhizobiales and Sphingomonadales have been associated with arid or desert soils [101,102]. Sphingomonadales are known to use naturally occurring compounds including root exudates [103] and Rhizobiales are responsible for nitrogen fixation in desert soils [104]. Alphaproteobacteria, despite their low frequency, play an essential role in biogeochemical cycles and nutrient transformations in soils [88].

In the present study, the majority of Chloroflexi and Bacteroidetes in soil are detected in equal proportions in the eDNA and iDNA pools. Chloroflexi shows a general adaptation to arid environments and is described as colonists of hot and cold hyperarid deserts [16,105,106] and Chitinophagaceae, affiliated to the phylum Bacteroidetes, is described as a cellulose and chitin-degrading taxa [107].

The relative abundance and diversity of archaea was comparably low in rocks and soils compared to the bacterial abundance and diversity (Figure 3). In the soil, Soil Crenarcheaotic Group occurred in equal parts in the e- and iDNA pools, which might be due to an active cell turnover. The Soil Crenarcheoatic Group are ammonia-oxidizing archaea linking cycling of nitrogen and carbon through nitrification and carbon fixation [108]. In general, archaea are absent or form a minor group of hypoliths [15,109]. To date, archaeal 16S rRNA signals have been discovered in cold deserts but little is known of their ecological role and their diversity and abundance are poorly understood [110].

In general, a larger proportion of unique OTUs in our study was found in soil and limestone compared to quartz-rich rocks (Figure 2d). This might refer to more favourable living conditions such as the presence of organic matter or temporary availability of water in soils and in limestone. Stomeo et al. [111] examined the influence of moisture sources on the lithic and sublithic niche and found an increased number of OTUs unique to the hypoliths within rainfall dominated locations. Slight differences in diversity and composition between the e- and iDNA pools of soil suggested a higher biomass turnover in soil compared to rocks. Sequence analyses referred to a potentially microbial active community consisting of Rubrobacter, Soil Crenarcheaotic Group, Rhizobiales, Sphingomonadales, and Chitinophagaceae in soil. This indicates that a sublithic habitat provides protection from harsh conditions such as UV-radiation, low water availability, or temperature extremes.

Our results show that rocks can represent unique extreme habitats which promote the growth of a variety of specialized microorganisms. Proteobacteria and Actinobacteria were the prominent viable representatives of different altered rocks but variations in the microbial communities were determined by changes in the minor population structure. Overall, the analytical separation in eDNA and iDNA has the potential to enhance the resolution of DNA data and avoids an overestimation of the diversity of the living microbial community. When considering the iDNA pool, the limited presence of the OTUs in the corresponding eDNA pool (particularly in quartz-rich rocks) might be an indicator of slow replication rates.

## 5. Conclusions

In the present study species adapted to the lithic niche were targeted by culture-independent and -dependent analyses. For the first time, crucial differences of the microbial community of rocks were determined by the extensive DNA separation method into eDNA and iDNA. By this approach we characterized the living organisms in the lithic microbial ecosystems of the Tsauchab Valley in Namibia as a form of terrestrial colonization in drylands and identified taxa as potentially active lithic microorganism.

Lithic and sublithic microbial communities were structural and compositional distinct. Both comprised bacterial taxa that are typical for arid environments including Actinobacteria and Alpha- and Gammaproteobacteria. The soil microbial community is dominated by a greater variety of microorganisms. Compared to rock samples, differences between eDNA and iDNA pools were less pronounced indicating a higher biomass turnover in soils. In addition, we could demonstrate that viable microorganism colonizing rocks and use rocks as a suitable lithobiontic ecological niche to facilitate life under harsh conditions.

## Figures and Tables

**Table 1:** Identification of isolated microorganisms. Physiological properties were selected to describe the characteristics of the lithic microbial community in arid areas based on literature. Based on Sanger sequencing, isolated from limestone (L) and quartz-rich sandstone (Q). P: phylum (A: Actinobacteria; F: Firmicutes; P: Proteobacteria); Nr.: Number of isolates; R: rock type.

Identification	P	Nr.	Rock	Physiological Characteristics
*Arthrobacter* sp.	A	3	L	Mineral cycling in soils [49]
*Kocuria* sp.	A	1	L	Rock-weathering bacteria, isolated from altered rocks, solubilize Si and Al [50]
*Lechevalieria* sp.	A	1	Q	Reported from arid habitats [51], Resistant to desiccation and high salinity [52]
*Microbacterium* sp.	A	3	Q	Involved in mineral weathering in soil and altered rocks [53,54]
*Streptomyces* sp.	A	7	L, Q	Resistant to low/high temperature [55], desiccation, salinity, [56,57,58]
*Bacillus* sp.	B	9	L, Q	Isolated from altered rocks and soils, rock-weathering species [49,50,59]. Found in Arid, desert and saline soils [2,11,60], Spore-forming bacteria [61], calcium carbonate precipitation [62]
*Massilia* sp.	P	2	L	Participate in carbonate rock dissolution by acid secretion [63]
*Microvirga* sp.	P	1	L	Part of the rhizobia and form nitrogen-fixing symbioses [64]

## Data Availability

Sequencing data were deposited as raw FASTQ files into the European Nucleotide Archive (sample accession: ERS3207779—ERS3207809).

## Supplementary Materials

The following are available online at https://www.mdpi.com/2076-2607/9/2/235/s1, Figure S1. Mineral composition of rock samples using XRD; Figure S2. Abundances of bacterial 16S rRNA gene copy numbers; Table S1. Sampling list including sample alias and location; Table S2. Detailed information on isolated microorganisms.
