# Supplementary material for: Living Lithic and Sublithic Bacterial Communities in Namibian Drylands
Source: Microorganisms. 2021 Jan 23;9(2):235. doi: 10.3390/microorganisms9020235 (PMC7911874; doi:10.3390/microorganisms9020235)
Supplement: Supplementary file 1 [file microorganisms-09-00235-s001.pdf]

microorganisms

MDPI

Supplementary Information

Living lithic and sublithic microbial communities in Namibian drylands

Steffi Genderjahn[a*], Simon Lewin[a,1], Fabian Horn[a], Anja M. Schleicher[b], Kai Mangelsdorf[c], Dirk Wagner[a,e]

[a] GFZ German Research Centre for Geosciences, Section Geomicrobiology, Telegrafenberg, Potsdam, 14473, Germany
[b] GFZ German Research Centre for Geosciences, Section Organic Geochemistry, Telegrafenberg, Potsdam, 14473, Germany
[c] GFZ German Research Centre for Geosciences, Section Anorganic Chemistry, Telegrafenberg, Potsdam, 14473, Germany
[d] University of Potsdam, Institute of Geosciences, 14476 Potsdam, Germany
[1] current address: Leibniz Centre for Agricultural Landscape Research, Section Microbial Biogeochemistry, Eberswalder Straβe 84, 15374, Müncheberg, Germany
* Correspondence: steffi.genderjahn@gfz-potsdam.de

Supplementary table legends:

Figure S1: Mineral composition of rock samples using XRD.

Figure S2: Abundances of bacterial 16S rRNA gene revealed by quantitative PCR from Tsauchab Valley, Namibia. Limestone, quartz-rich shale and quartz-rich sandstone were quantified by three intersections across their weathering profile from unaltered core over the subsurface to the surface and compared to adjacent soil. Standard deviations are indicated by an error bar

Table S1: Sampling list including sample alias and location.

Table S2: Identification of microorganisms from limestone and quartz-rich sandstone based on Sanger sequencing. Medium R2S for facultative heterotrophic aerobes and SSM for mineral solubilizing bacteria (sucrose salt medium). N: Number of isolates

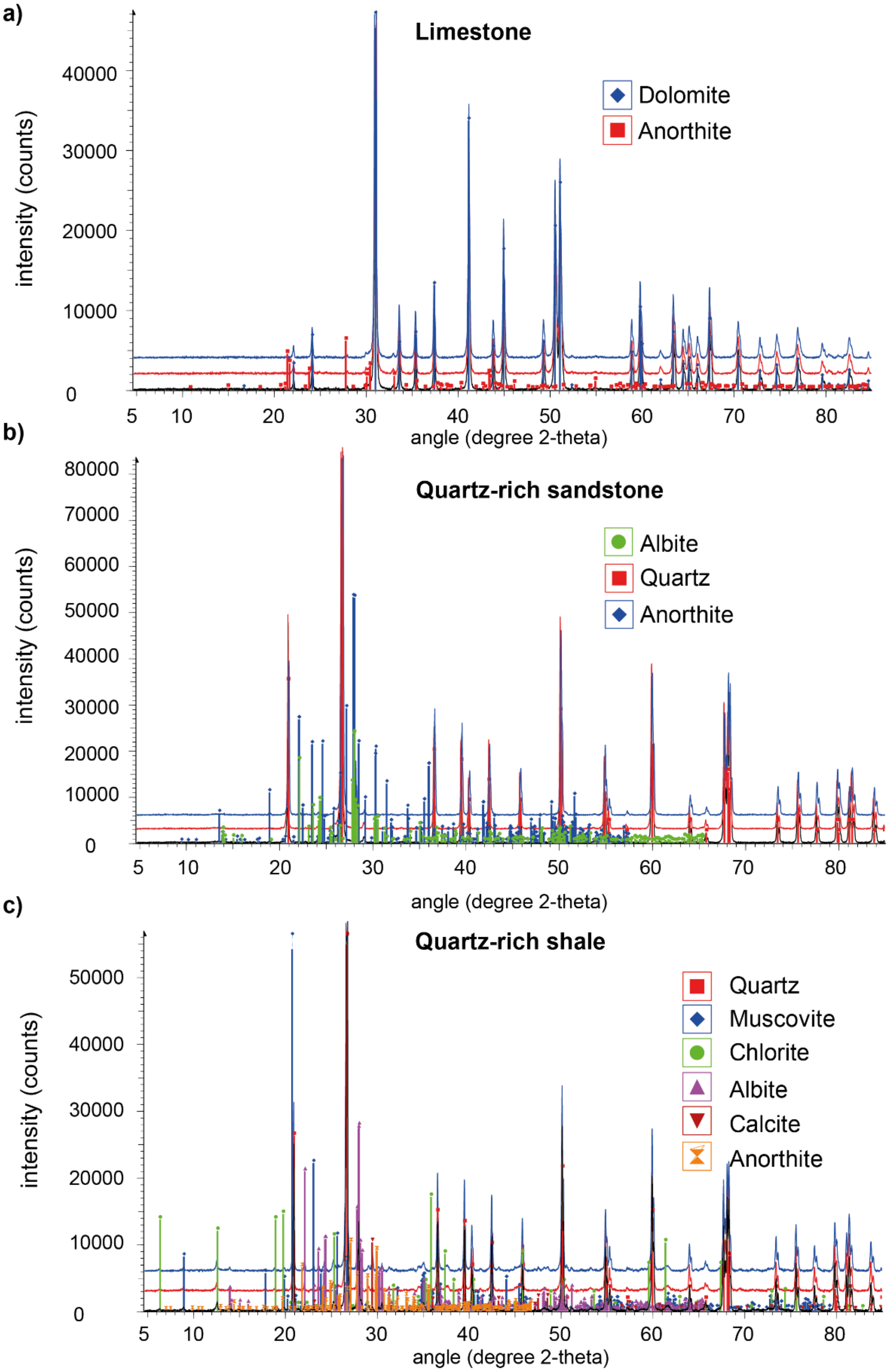

Figure S1: Mineral composition of rock samples using XRD.

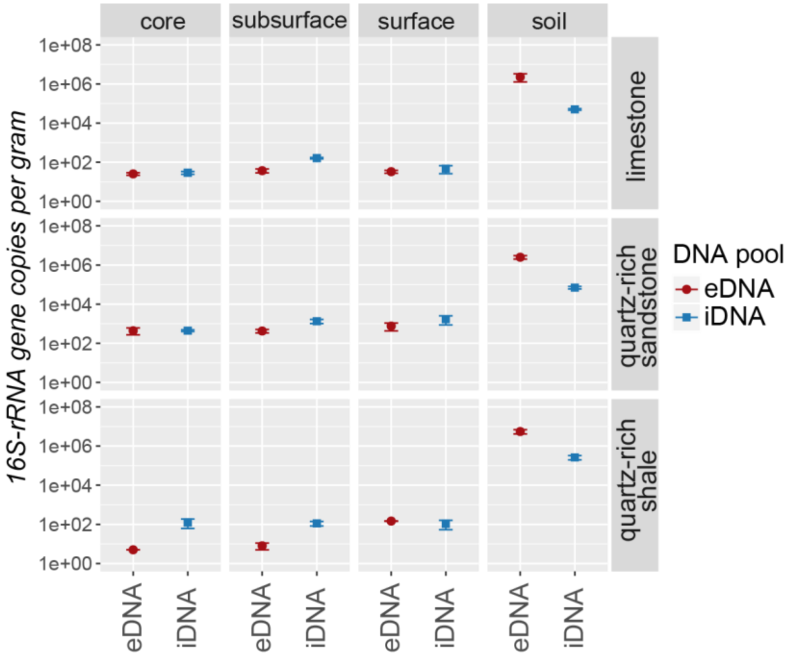

Figure S2: Abundances of bacterial 16S rRNA gene revealed by quantitative PCR from Tsauchab Valley, Namibia. Limestone, quartz-rich shale and quartz-rich sandstone were quantified by three intersections across their weathering profile from unaltered core over the subsurface to the surface and compared to adjacent soil. Standard deviations are indicated by an error bar.

Table S1: Sampling list including sample alias and location.

Project	03G0861B
short title	Lithic microbial communities of dryland samples
Collection date	18.03.2017
location	Namibia
geographic location	24°26,37′S, 016°10,31′E
geographic elevation	1160 m
geographic region	Tsauchab River Valley, Namibia

**sample alias**	**DNA pool**	**material**	**section**
A1103	eDNA	limestone	surface
A1201	eDNA	limestone	core
A1202	eDNA	limestone	subsurface
A1203	eDNA	limestone	surface
A1401	eDNA	quartz-rich sandstone	core
A1402	eDNA	quartz-rich sandstone	subsurface
A1403	eDNA	quartz-rich sandstone	surface
A1501	eDNA	quartz-rich sandstone	core
A1502	eDNA	quartz-rich sandstone	subsurface
A1503	eDNA	quartz-rich sandstone	surface
A1601	eDNA	quartz-rich shale	core
A1602	eDNA	quartz-rich shale	subsurface
A1603	eDNA	quartz-rich shale	surface
B1101	iDNA	limestone	core
B1202	iDNA	limestone	subsurface
B1203	iDNA	limestone	subsurface
B1402	iDNA	quartz-rich sandstone	subsurface
B1403	iDNA	quartz-rich sandstone	core
B1601	iDNA	quartz-rich shale	core
B1602	iDNA	quartz-rich shale	subsurface
B1602	iDNA	quartz-rich shale	subsurface
A1106	eDNA	soil	limestone
A1206	eDNA	soil	limestone
A1406	eDNA	soil	quartz-rich sandstone
A1506	eDNA	soil	quartz-rich sandstone
A1606	eDNA	soil	quartz-rich shale
B1106	iDNA	soil	limestone
B1206	iDNA	soil	limestone
B1406	iDNA	soil	quartz-rich sandstone
B1506	iDNA	soil	quartz-rich sandstone
B1606	iDNA	soil	quartz-rich shale
X00000	negative control		
Z11111	positive control	E. coli	

microorganisms

MDPI

Table S2: Identification of microorganisms from limestone and quartz-rich sandstone based on Sanger sequencing. Medium R2S for facultative heterotrophic aerobes and SSM for mineral solubilizing bacteria (sucrose salt medium). N: Number of isolates

	Phylum	Identification	Medium	N	% Identity	GenBank accession number
Limestone		*Protaetiibacter* sp.	R2A	1	97	CP059987
		Arthrobacter sp.	R2A, SSM	2	98 – 99	CP019304
		Kocuria rosea	R2A	1	98	LR134391
		Streptomyces sp.	R2A, SSM	2	99 – 100	KC336160, CP050504
		Streptomyces ansochromogenes	SSM	1	100	AB184448
		Bacillus axarquiensis	R2A	1	98	KY608836
		Bacillus paralicheniformis	R2A	2	99	CP033198
		Bacillus niacini	R2A	1	98	BCVA01000102
		Microvirga sp.	R2A	1	99	CP029481
		Massilia sp.	R2A	2	97 – 98	CP012640
Quartz-rich sandstone		*Microbacterium* sp.	R2A, SSM	3	99 – 100	CP043430
		Arthrobacter sp.	R2A	1	98	AY618581
		Lechevalieria sp.	R2A	1	99	FJ911538
		Streptomyces sp.	SSM	3	99 – 100	LC429299, KC336160, CP050504
		Streptomyces reticuli	SSM	1	100	LN997842
		Bacillus subtilis	R2A, SSM	4	99 – 100	MN704542, MT643824, MT641205, CP018184
		Bacillus tequilensis	R2A	1	99	MT326211

Actinobacteria   Firmicutes   Proteobacteria

microorganisms
